# Parahydrogen-Induced Hyperpolarization of Unsaturated Phosphoric Acid Derivatives

**DOI:** 10.3390/ijms24010557

**Published:** 2022-12-29

**Authors:** Veronika V. Zlobina, Alexey S. Kiryutin, Igor A. Nikovskiy, Oleg I. Artyushin, Vitaly P. Kozinenko, Alexander S. Peregudov, Alexandra V. Yurkovskaya, Valentin V. Novikov

**Affiliations:** 1 Nesmeyanov Institute of Organoelement Compounds, Russian Academy of Sciences, Vavilova Str. 28, 119991 Moscow, Russia; 2 Moscow Institute of Physics and Technology, National Research University, Institutskiy per. 9, 141700 Dolgoprudny, Russia; 3 International Tomography Center, Siberian Branch of the Russian Academy of Sciences, Institutskaya Str. 3A, 630090 Novosibirsk, Russia; 4 Department of Physics, Novosibirsk State University, Pirogova Str. 2, 30090 Novosibirsk, Russia; 5 BMSTU Center of National Technological Initiative “Digital Material Science: New Material and Substances”, Bauman Moscow State Technical University, 2nd Baumanskaya Str. 5, 105005 Moscow, Russia

**Keywords:** heteroatomic nuclei, hyperpolarization, hyperpolarized probe, magnetic resonance imaging, NMR spectroscopy, ^31^P nucleus, parahydrogen, polarization transfer, phosphates, signal amplification

## Abstract

Parahydrogen-induced nuclear polarization offers a significant increase in the sensitivity of NMR spectroscopy to create new probes for medical diagnostics by magnetic resonance imaging. As precursors of the biocompatible hyperpolarized probes, unsaturated derivatives of phosphoric acid, propargyl and allyl phosphates, are proposed. The polarization transfer to ^1^H and ^31^P nuclei of the products of their hydrogenation by parahydrogen under the ALTADENA and PASADENA conditions, and by the PH-ECHO-INEPT+ pulse sequence of NMR spectroscopy, resulted in a very high signal amplification, which is among the largest for parahydrogen-induced nuclear polarization transfer to the ^31^P nucleus.

## 1. Introduction

Nuclear magnetic resonance (NMR) spectroscopy is one of the most powerful analytical methods in chemistry [1], biology [2] and medicine [3]. However, it often suffers from a low sensitivity owing to an extremely small difference in the populations of the nuclear spin states, which are as low as 10^−5^–10^−4^ in the magnetic fields of several Tesla at room temperature, the conditions typically employed in modern NMR spectrometers [4,5]. Overcoming this limitation [6,7] can revolutionize quantitative visualization of metabolic processes inside a living organism [8] and early diagnosis of associated pathologies by magnetic resonance imaging (MRI) [9]. To do so, a nonequilibrium polarization of the nuclei can be achieved by transferring the spin order from the parahydrogen to the investigated molecule—referred to as parahydrogen-induced nuclear polarization (PHIP) [10]. Unlike other ways of generating nuclear hyperpolarization, such as noble gas optical pumping [11,12,13] or dynamic nuclear polarization [14,15], PHIP produces a wide range of polarized molecules, including MRI contrast agents [16], with no need for expensive equipment. The transfer of the spin order from the protons of the substrate molecule to its other nuclei [17,18] further increases the sensitivity and resolution of MRI, as the signals in the heteronuclear NMR spectra are less prone to overlap.

Heteroatomic nuclei often feature longer relaxation times and, therefore, longer polarization lifetimes [19]. Among them, the ^31^P nucleus is highly promising for medical applications, as phosphorus compounds are involved in many biological reactions. Its large gyromagnetic ratio and the spin of ½ are behind the high sensitivity of PHIP-assisted NMR spectroscopy [20,21].

**Scheme 1:**
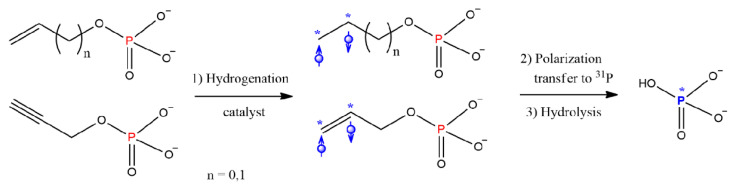
A PHIP pathway towards hyperpolarized phosphate. Polarized spins indicated by symbol *.

Inorganic phosphate, which is the most common phosphorous-containing compound in our bodies (from our DNA to the bones), is an attractive hyperpolarized probe for MRI [22] that can be created in three steps (Scheme 1). The first step is the hydrogenation of a suitable precursor compound, such as allyl or propargyl phosphate, by the parahydrogen. These phosphoric acid esters are chemically stable and contain an unsaturated fragment close to the phosphorus nuclei available for hydrogenation.

In the next step, the polarization is transferred from the hydrogenated fragment to the ^31^P nucleus. This could be carried out in several ways, including the spontaneous transfer in a strong magnetic field, caused by the nuclear Overhauser effect [23], or in a weak magnetic field, owing to spin–spin interactions between the protons and the heteroatomic nucleus (adiabatic longitudinal transport after dissociation engenders net alignment, ALTADENA) [24]. Experimentally, this is done by performing the hydrogenation of the precursor compound, either inside the NMR spectrometer (PASADENA, parahydrogen and synthesis allow dramatic enhancement of nuclear alignment), or in a weak field outside of it, with the following adiabatic transfer of the sample to the spectrometer’s probe for the spectra registration (ALTADENA). Another option is the stimulated transfer that is induced by radio-frequency pulse sequences, such as INEPT (insensitive nuclei enhancement by polarization transfer) [25]. The final step is the polarization transfer to the ^31^P nuclei and the hydrolysis of a phosphoric acid derivative that results in a hyperpolarized inorganic phosphate (Scheme 1).

Here, this three-step approach is applied to the unsaturated derivatives of phosphoric acid, allyl and propargyl phosphates, to produce biocompatible probes for MRI with very long relaxation times.

## 2. Results and Discussion

### 2.1. Parahydrogen Hydrogenation and Identification of the Products

**Scheme 2:**
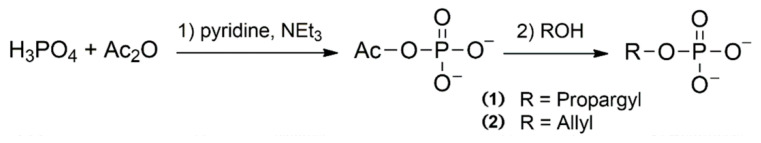
Phosphate synthesis.

Two unsaturated derivatives of phosphoric acid were chosen as precursor compounds for the PHIP-assisted NMR spectroscopy; these are propargyl and allyl phosphates of cyclohexylammonium that were synthesized by esterification of phosphoric acid by the corresponding alcohol in the presence of acetic anhydride (Scheme 2).

**Figure 1:**
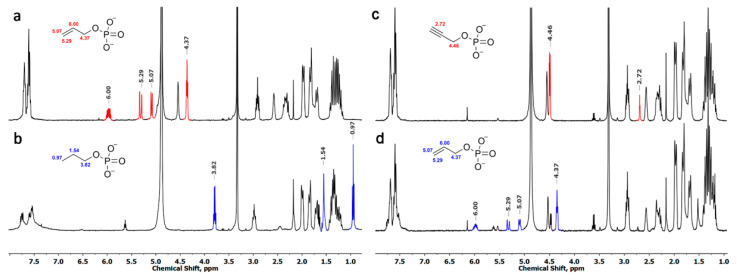
Shows ^1^H NMR spectra of cyclohexylamine allyl phosphate **2** before (**a**) and after (**b**) hydrogenation, and of cyclohexylamine propargyl phosphate **1** before (**c**) and after (**d**) hydrogenation. To avoid the phase distortion and to simplify the comparison of the signal intensities, the spectra were registered after the hyperpolarization relaxed to the thermal polarization levels.

The obtained phosphates were then hydrogenated by bubbling parahydrogen through their solutions in deuterated methanol in the presence of the complex [Rh(dppb)(COD)]BF_4_ as a catalyst [26] inside and outside of the NMR spectrometer. The collected NMR spectra featured intensive signals of the hydrogenation products together with an expected [27,28] signal of orthohydrogen (chemical shift δ = 4.57 ppm) [29] (Figures S1 and S2). As follows from these spectra, the allyl phosphate **2** converts into the propyl-containing product upon hydrogenation (Figure 1a,b), which, in the case of propargyl phosphate **1,** occurs stepwise via the formation of an intermediate allyl phosphate (Figure 1c,d).

### 2.2. Hydrogenation under the ALTADENA Conditions

**Figure 2:**
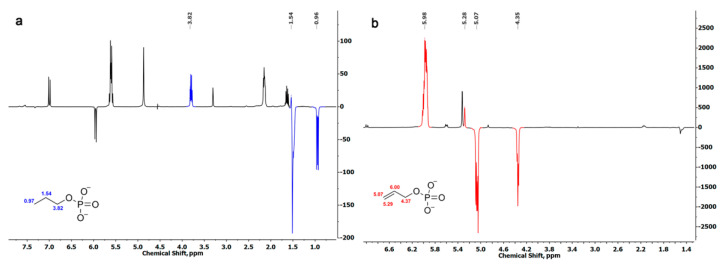
Shows ^1^H NMR ALTADENA spectra obtained by hydrogenation of cyclohexylamine allyl phosphate **2** (**a**) and cyclohexylamine propargyl phosphate **1** (**b**).

In the ALTADENA [24] experiment, the hydrogenation of the phosphates **1** and **2** was performed in a weak field of 2 mT followed by the adiabatic transfer of the sample into the strong field of the NMR spectrometer for spectra acquisition. The field strength of 2 mT was chosen as the smallest available one outside the magnetic shield. Going to a smaller field, e.g., in a submicrotesla region, might have caused a polarization transfer to the ^31^P nuclei. The resulting ALTADENA spectra demonstrated an expected peculiar line shape of the signals with the phase inversion [30]. If the hydrogenation occurs in a weak magnetic field (e.g., Earth’s magnetic field), the difference in the chemical shifts of the two protons after their binding to the substrate is smaller than the spin–spin interaction between them (|*γ*∆*δB*0| < |2*πJ*|). Upon the adiabatic transfer of the hydrogenation product into a strong magnetic field, one of the states |*αβ*〉 or |*βα*〉, depending on the sign of the spin–spin interaction constant, becomes populated. As a result, the NMR spectrum features the signals with a phase shifted by 180 degrees (Figure 2).

For the phosphates **2** and **1**, the PHIP-based signal amplification, which is the ratio of the relative integral intensity of the signals from the polarized hydrogenation products to the relative integral intensity of the same signals after the polarization relaxation, reaches as high as 10 and 3276, respectively.

### 2.3. Hydrogenation under the PASADENA Conditions

**Figure 3:**
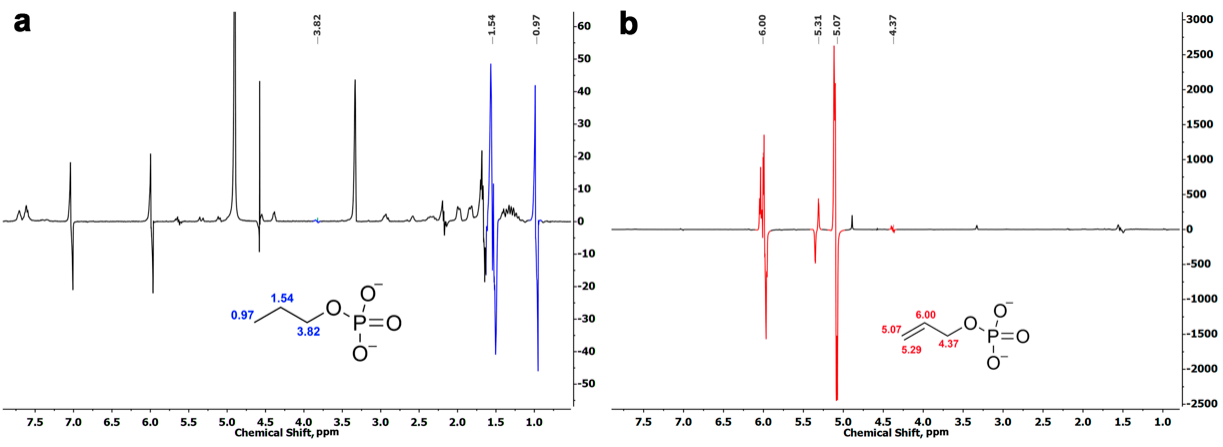
Shows ^1^H NMR PASADENA spectra of cyclohexylamine allyl phosphate **2** (**a**) and cyclohexylamine propargyl phosphate **1** (**b**). The strong antiphase signals at 6 and 7 ppm in the spectra of cyclohexylamine allyl phosphate (**a**) belong to the known non-reactive impurities in the bubbling system.

If the hydrogenation occurs in the strong field of the NMR spectrometer (the PASADENA conditions [23]), the affected signals in the NMR spectra show an antiphase behavior (Figure 3). The difference in the chemical shifts of the protons bonded to the substrate is much larger than the spin–spin coupling constant (|*γ*∆*δB*0| ≫ |2*πJ*|). As a result, the spin order of the parahydrogen molecule transforms into the population of the states |*αβ*〉 and |*βα*〉 of the hydrogenated product, thereby causing strongly enhanced antiphase multiplets to appear in the NMR spectra.

As in the ALTADENA experiment above, the signal amplification under the PASADENA conditions, which was estimated by comparing the signal intensity before and after the relaxation of the polarization, was much higher for propargyl phosphate **1** than for allyl phosphate **2** (1588 and 6, respectively). The higher values of this amplification under the ALTADENA conditions stem from the difference in the nuclear relaxation rates in low and high magnetic fields [31].

### 2.4. Spontaneous Polarization Transfer

**Figure 4:**
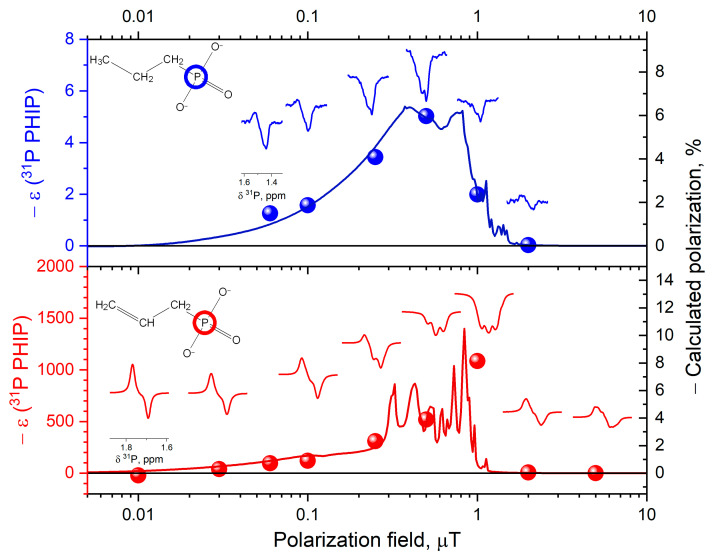
The signal amplification and polarization levels of the ^31^P nuclei upon the polarization transfer by magnetic field-dependent hydrogenation by parahydrogen. The blue and red circles are the experimental values of the ^31^P signal amplifications, and the solid lines show the best fits by the numerical model that employed spin–spin coupling and chemical shifts from high-field ^1^H and ^31^P NMR spectra (see SI) and neglected the relaxation effects. The polarization scale of the calculated curves is adjusted to guide the eye.

The possibility of the spontaneous polarization transfer to the ^31^P nucleus was probed by hydrogenating the phosphates **1** and **2** in the magnetic field that varied from nearly 0 to 2000 nT. For both these phosphates, a line shape of the ^31^P signal was clearly field-dependent (Figure 4). The largest amplification of the signal in the ^31^P NMR spectrum for phosphates **2** and **1** of 5 and 1100, respectively, was observed at the magnetic fields of 500 and 1000 nT (Figure 4). The value for propargyl phosphate **1** is among the highest observed for PHIP-assisted ^31^P signal amplification [22,32]. To validate the coherent nature of polarization transfer in these experiments, we performed numerical simulation of the observed ^31^P PHIP field dependence as described in the Methods section.

### 2.5. Polarization Transfer with the INEPT Pulse Sequence

An alternative approach for transferring the polarization from parahydrogen to a heteroatomic nucleus [1] is to use a PH-INEPT pulse sequence [30] of conventional NMR spectroscopy. As the polarized allyl and propyl phosphates have no direct J-coupling between the polarized protons and ^31^P nuclei, we employed a PH-ECHO-INEPT+ [33,34] sequence which, in contrast to PH-INEPT+ [30,35], has an important additional step, an echo on a proton channel for the polarization transfer to the methylene protons of an intermediate. Three corresponding delays of the pulse sequence were optimized to maximize the efficiency of the polarization transfer. This technique was here applied to propargyl phosphate **1**, as allyl phosphate **2** allowed only relatively low amplifications to be achieved under both the PASADENA and ALTADENA conditions. For propargyl phosphate **1**, the PH-ECHO-INEPT+ polarization transfer resulted in the signal amplification of 1672 (Figure S3). The use of the PH-ECHO-INEPT+ sequence allowed additionally increasing the efficiency of the polarization transfer, owing to longer relaxation times of phosphorus nuclei in a high field as compared to low-field conditions, so that the hyperpolarization is not dissipated during the sample transfer between the field regions. Although the resulting amplification is much smaller than the value of 3588 obtained by polarization transfer under SABRE (signal amplification by reversible exchange) conditions to a deuterated pyridine-containing phosphonate ligand [20], the amplification of the ^31^P signal is among the largest achieved by PHIP [21,36].

### 2.6. Hydrolysis

The proposed pathway towards the hyperpolarized phosphate (Scheme 1) implied the hydrolytic cleavage of the formerly unsaturated fragment from the ^31^P nucleus. Unfortunately, both phosphates **1** and **2** resisted the hydrolysis at all the probed pH values, from 1 to 14. They showed no signs of degradations in water over two weeks, as judged by the NMR spectra collected before and after this time. The strategy to achieve improved hydrolytic activity of hydrogenated phosphates **1** and **2** by decorating them with hydrolysis-enhancing groups is now being explored in our group.

## 3. Materials and Methods

**Synthesis.** All synthetic manipulations were carried out in a nitrogen atmosphere unless stated otherwise. Solvents were purchased from commercial sources and purified by distilling from conventional drying agents under an argon atmosphere prior to use.

**Cyclohexylammonium allyl phosphate (2).** To a mixture of crystalline phosphoric acid (2.0 g, 20.4 mmol) and pyridine (8.5 mL, 104.5 mmol) under stirring, allyl alcohol (11.9 g, 205 mmol) and triethylamine (5.5 mL, 41 mmol) were added via a dropping funnel. After the complete dissolution of solids, acetic anhydride (4 mL, 42.3 mmol) was added dropwise. The reaction mixture was stirred for 2 h at 90 °C and then cooled to r.t. After addition of water (10 mL), the reaction mixture was stirred at 90 °C for 1 h and cooled to r.t. The solution was diluted with water (25 mL). The aqueous phase was washed 3 times with diethyl ether (50 mL) and concentrated; the oily liquid was dissolved in acetone/water (9:1), and then cyclohexylamine (4.2 mL, 61 mmol) was added. The mixture was cooled at 4 °C and left at this temperature for 12 h to produce a white crystalline solid, which was collected by filtration and dried. The solid was then heated in ethanol, the insoluble residue was filtered off and the filtrate was cooled for 12 h at 4 °C. The white solid was filtered, washed with ethanol and dried under vacuum [37]. Yield: 2.1 g (66%). Found (%): C 53.39; H 10.11; N 8.47. Calculated C_15_H_33_N_2_O_4_P (%): C 53.55; H 9.98; N 8.33. ^1^H NMR (CD_3_OD) before hydrogenation, δ (ppm) = 4.37 (m, 2H, 3H-allyl), 5.07 (dd, 1H, 1H-allyl, ^3^J = 10.5 Hz, ^2^J = 1.7 Hz), 5.29 (dd, 1H, 1H-allyl, ^3^J = 17.2 Hz, ^2^J = 1.7 Hz), 6.00 (m, 1H, 2H-allyl); after hydrogenation, δ (ppm) = 0.97 (t, 3H, 1H-propyl, ^3^J = 7.4 Hz), 1.54 (s, 2H-propyl), 1.82 (dd, 1H, 1H-allyl, ^3^J = 6.6 Hz). ^31^P NMR (DMSO) before hydrogenation, δ (ppm) = 2.55 (Figure S4).

**Cyclohexylammonium propargyl phosphate (1).** Phosphorous acid (1.6 g, 19.6 mmol) was dissolved in a mixture of propargyl alcohol (38.5 g, 686.7 mmol) and triethylamine (10 mL). The resulting mixture was stirred for 5 min while adding iodine (7.6 g, 30.0 mmol), and then it was added to a mixture of acetone (400 mL) and triethylamine (15 mL). After stirring for 2 h at r.t., cyclohexylamine (30 mL) was added. The colorless precipitate was filtered and recrystallized from ethanol with a few drops of cyclohexylamine [38]. Yield: 2.05 g. (64%). Found (%): C 53.69; H 9.24; N 8.48. Calculated C_15_H_31_N_2_O_4_P (%): C 53.88; H 9.34; N 8.38. ^1^H NMR (CD_3_OD) before hydrogenation, δ (ppm) = 2.72 (t, 1H-propargyl, ^4^J = 2.5 Hz), 4.46 (dd, 3H-allyl, ^4^J = 2.5 Hz, ^3^J_H-P_ = 6.3 Hz); after hydrogenation, δ (ppm) = 4.37 (m, 2H, 3H-allyl), 5.07 (dd, 1H, 1H-allyl, ^3^J = 10.5 Hz, ^2^J = 1.7 Hz), 5.29 (dd, 1H, 1H-allyl, ^3^J = 17.2 Hz, ^2^J = 1.7 Hz), 6.00 (m, 1H, 2H-allyl).). ^31^P NMR (DMSO) before hydrogenation, δ (ppm) = 2.49 (Figure S5).

**NMR spectroscopy.** The ^1^H и ^31^P NMR spectra were collected with a Bruker Avance III HD 400 spectrometer (proton frequency 400 MHz, field strength 9.4 T) and a Bruker Avance 300 spectrometer (proton frequency 300 MHz, field strength 7.05 T).

**Hydrogenation by parahydrogen.** To produce parahydrogen for hydrogenation, the parahydrogen generator CFA-200-H2CELL (CryoPribor, Cryotrade Engineering, Russia), based on a Cryocooler Zephyr HC-4A (Sumitomo, Japan) that provides parahydrogen enrichment of more than 95%, was used. Parahydrogen was bubbled through the solutions of the precursor compounds with a home-built fully automated setup [39,40]; the bubbling pressure was 4 atm. NMR spectra were obtained by sequential recording of 16 spectra with a parahydrogen bubbling time of 10 s.

**Hydrogenation under the ALTADENA conditions**. NMR experiments with fast magnetic field cycling and PHIP were performed with a home-built setup based on a Bruker Avance HD 400 MHz NMR spectrometer [40,41]. The setup includes a magnetic shield placed on top of the bore of the spectrometer’s superconducting magnet that allows ultralow magnetic fields as low as 5 nT to be achieved. The desired value of the ultralow magnetic field is set by adjusting the current in the induction coils located inside the magnetic shield. The sample is mechanically transferred between the detection zone of the spectrometer and the magnetic shield via a plastic rail driven by a stepper motor. An additional Z coil was used, controlled through a relay to alternate between 50 μT (Earth’s magnetic field) and the ultralow field. This type of field variation, which is necessary to effectuate an abrupt field switch between several mT and nT, has a duration of 100 μs; shuttling the sample from the spectrometer field to the magnetic shield takes about 0.5 s [42]. Hydrogenation at an arbitrary magnetic field was performed using the above home-built automated gas-flow system [34] that allows controlling the timing of both the parahydrogen bubbling and the magnetic field switching from the spectrometer console. The parahydrogen bubbling pressure was set to 3 bar with a 0.1-bar differential between the inlet and outlet of the tube, thereby providing an optimal gas flow rate of about 20–40 cm^3^/min. Temperature of the sample was 25 °C.

**Hydrogenation under the PASADENA conditions**. PASADENA experiments were carried out in situ in an NMR magnet in the same way as described above, but without the magnetic field cycling.

**Polarization transfer via field selection.** Polarization of the ^31^P nuclei was performed using the above home-built automated field cycling setup, with addition of magnetic shield and electromagnets inside to vary the magnetic field from 50 nT to 2 mT. The details of the setup can be found elsewhere [40,41].

The numerical simulation of the field dependence was performed by using an approach described in [42]. In a nutshell, it assumes that the coherences arising due to spin mixing at a chosen magnetic field (Table S1 and Scheme S1) are averaged to zero over the hydrogenation period. To obtain the resulting density matrix, we transformed the initial density matrix of parahydrogen protons into the eigenbasis of the spin Hamiltonian at a chosen field and set all the off-diagonal elements to zero. The level of ^31^P polarization was obtained by calculating the expectation value of the ${\hat{I}}_{z}$ spin operator corresponding to the ^31^P nucleus.

**PH-ECHO-INEPT+.** A PH-ECHO-INEPT+ experiment was carried out with a Bruker 400 MHz spectrometer. Parahydrogen was bubbled through the sample inside the spectrometer for 10 s. Bubbling was followed by a delay—the waiting time required to get rid of bubbles and allow the sample to return to the coil volume inside the NMR sample tube. Following this delay, the frequency-selective PH-ECHO-INEPT+ polarization transfer pulse sequence was applied. For the optimal polarization transfer, the following delays in the pulse sequence were used: 1 = 26 ms, 2 = 42 ms, 3 = 20 ms. The physical meaning of these delays and the detailed description of the pulse sequence are provided in [33].

## 4. Conclusions

Two unsaturated derivatives of phosphoric acid, propargyl and allyl phosphates, were probed as the precursors of the biocompatible hyperpolarized probe for MRI. Their hydrogenation by parahydrogen, followed by the polarization transfer triggered by PASADENA and ALTADENA conditions and by a PH-ECHO-INEPT+ sequence of NMR spectroscopy, allowed a very high enhancement of the signals of the protons (up to 3000) and of the ^31^P nucleus (up to 1700) in the corresponding NMR spectra to be achieved. For propargyl phosphate, it is among the highest for the polarization transfer to the ^31^P nucleus induced by PHIP. Unfortunately, the hydrogenation products of both the phosphates were extremely stable towards hydrolysis, thereby preventing us from obtaining a hyperpolarized inorganic phosphate, a biocompatible probe for MRI with very long relaxation times [19,43]. Further efforts to obtain the derivatives of phosphoric acid that can be polarized by parahydrogen and hydrolyzed after the hydrogenation may pave the way towards using the available hyperpolarized inorganic phosphate as a routine tool in medical diagnostics by MRI.

## Supplementary Materials

The following supporting information can be downloaded at: https://www.mdpi.com/article/10.3390/ijms24010557/s1. Reference [44] is cited in the supplementary materials.
