# Supplementary material for: Parahydrogen-Induced Hyperpolarization of Unsaturated Phosphoric Acid Derivatives
Source: Int J Mol Sci. 2022 Dec 29;24(1):557. doi: 10.3390/ijms24010557 (PMC9820518; doi:10.3390/ijms24010557)
Supplement: Supplementary file 1 [file ijms-24-00557-s001.zip › ijms-2111562-supplementary.pdf]

Supporting Information

Parahydrogen-Induced Hyperpolarization of Unsaturated Phosphoric Acid Derivatives

Veronika V. Zlobina[1,2], Alexey S. Kiryutin[3,4], Igor A. Nikovskiy[1], Oleg I. Artyushin[1], Vitaly P. Kozinenko[3,4], Alexander S. Peregudov[1], Alexandra V. Yurkovskaya[3,4], Valentin V. Novikov[2,5*]

[1]Nesmeyanov Institute of Organoelement Compounds, Russian Academy of Sciences, Vavilova Str. 28, 119991 Moscow, Russia

[2]Moscow Institute of Physics and Technology, National Research University, Institutskiy per. 9, Dolgoprudny, 141700 Moscow Region, Russia

[3]International Tomography Center, Siberian Branch of the Russian Academy of Sciences, Institutskaya Str. 3A, 630090 Novosibirsk, Russia

[4]Novosibirsk State University, Department of Physics, Pirogova Str. 2, 30090 Novosibirsk, Russia

[5]BMSTU Center of National Technological Initiative "Digital Material Science: New Material and Substances", Bauman Moscow State Technical University, 2nd Baumanskaya Str. 5, 105005 Moscow, Russia

[*]Correspondence: novikov84@ineos.ac.ru

	Page number
**Figure S1.** ^{1}H NMR spectrum of parahydrogen-polarized allyl phosphate cyclohexylamine	**3**
**Figure S2.** ^{1}H NMR spectrum of parahydrogen-polarized propargyl phosphate cyclohexylamine	**4**
**Figure S3.** ^{31}P NMR INEPT spectrum of propargyl phosphate cyclohexylamine	**5**
**Figure S4.** ^{31}P NMR spectrum of allyl phosphate cyclohexylamine	**6**
**Figure S5.** ^{31}P NMR spectrum of propargyl phosphate cyclohexylamine	**7**
**Table S1.** Spin-spin scalar couplings and chemical shifts from NMR spectra of allyl phosphate and propargyl phosphate.	**8**
**Scheme S1.**	**8**
**Supplementary references**	**8**

Supplementary Figures:

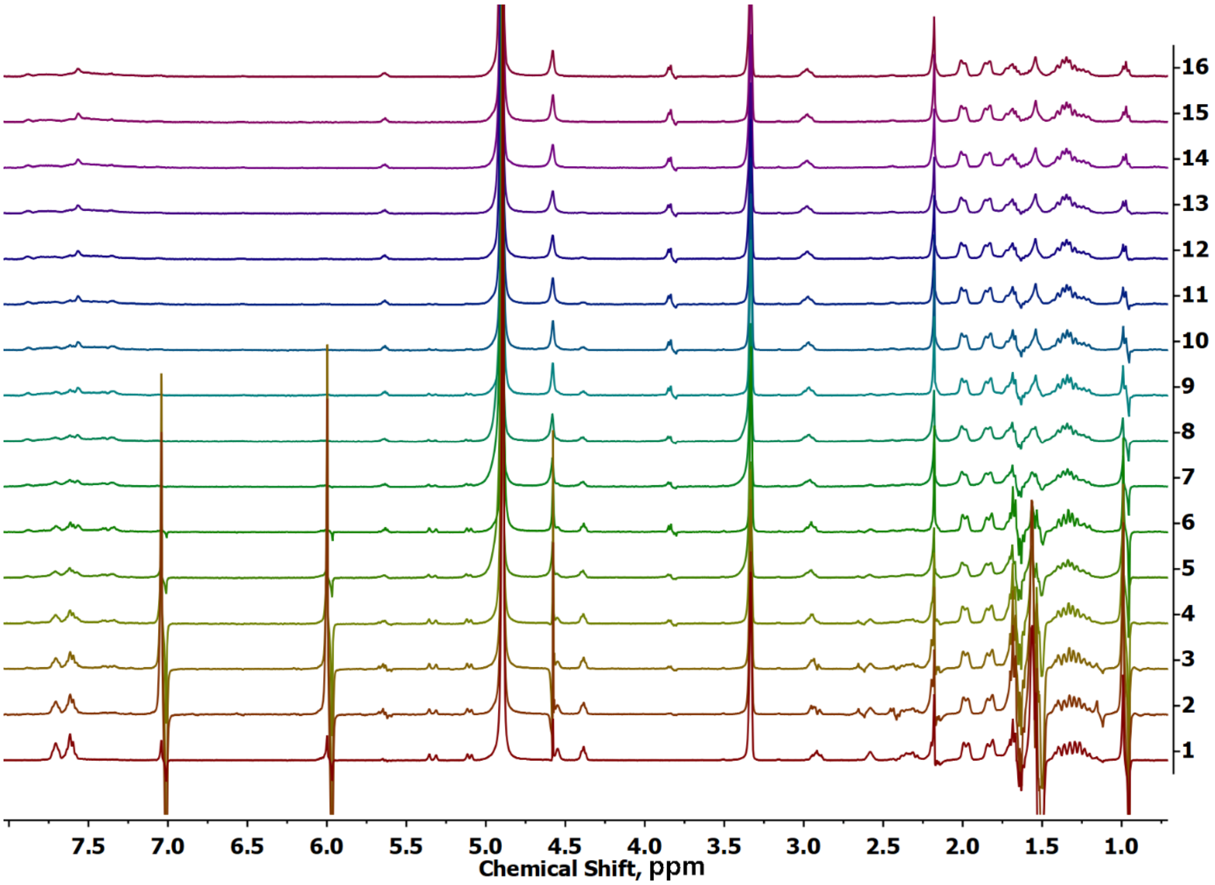

Figure S1. ^{1}H NMR spectrum of parahydrogen-polarized allyl phosphate cyclohexylamine.

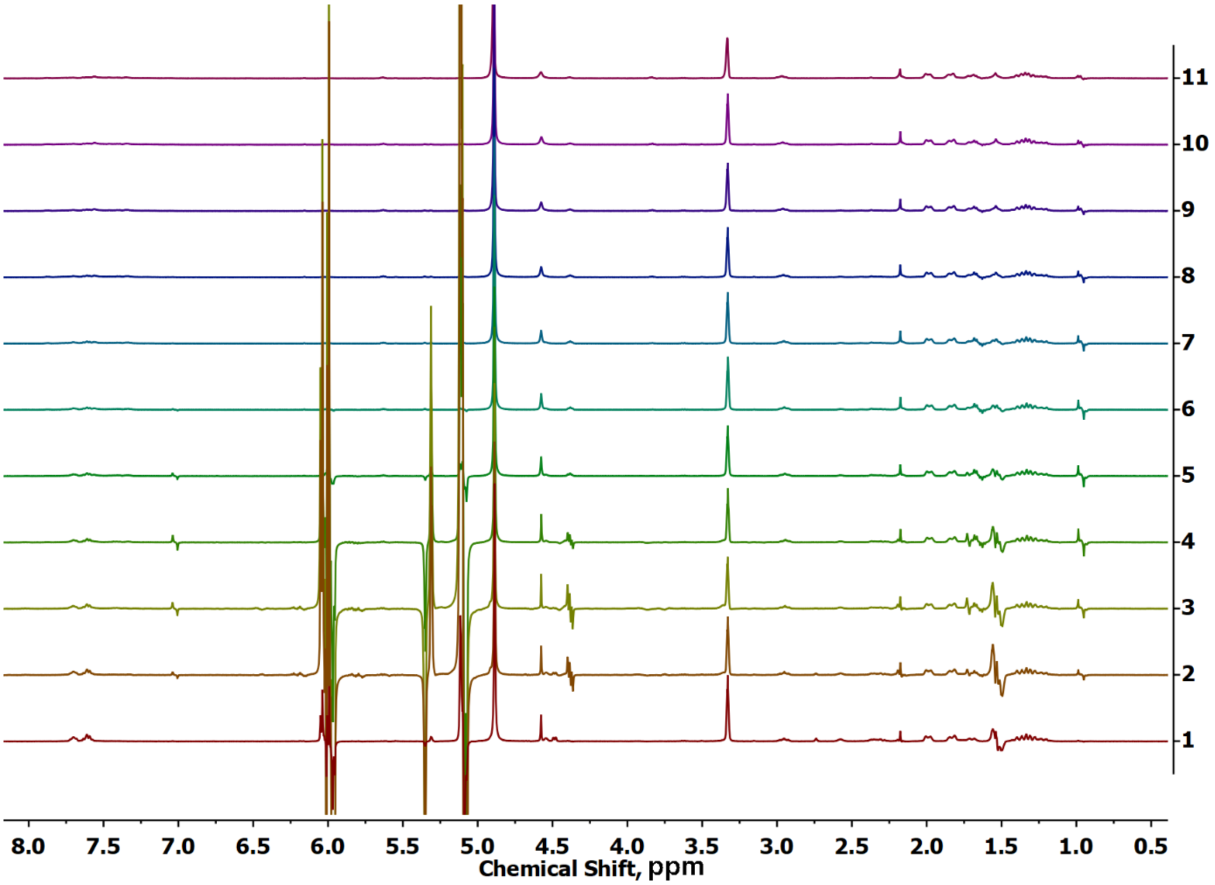

Figure S2. ^{1}H NMR spectrum of parahydrogen-polarized propargyl phosphate cyclohexylamine.

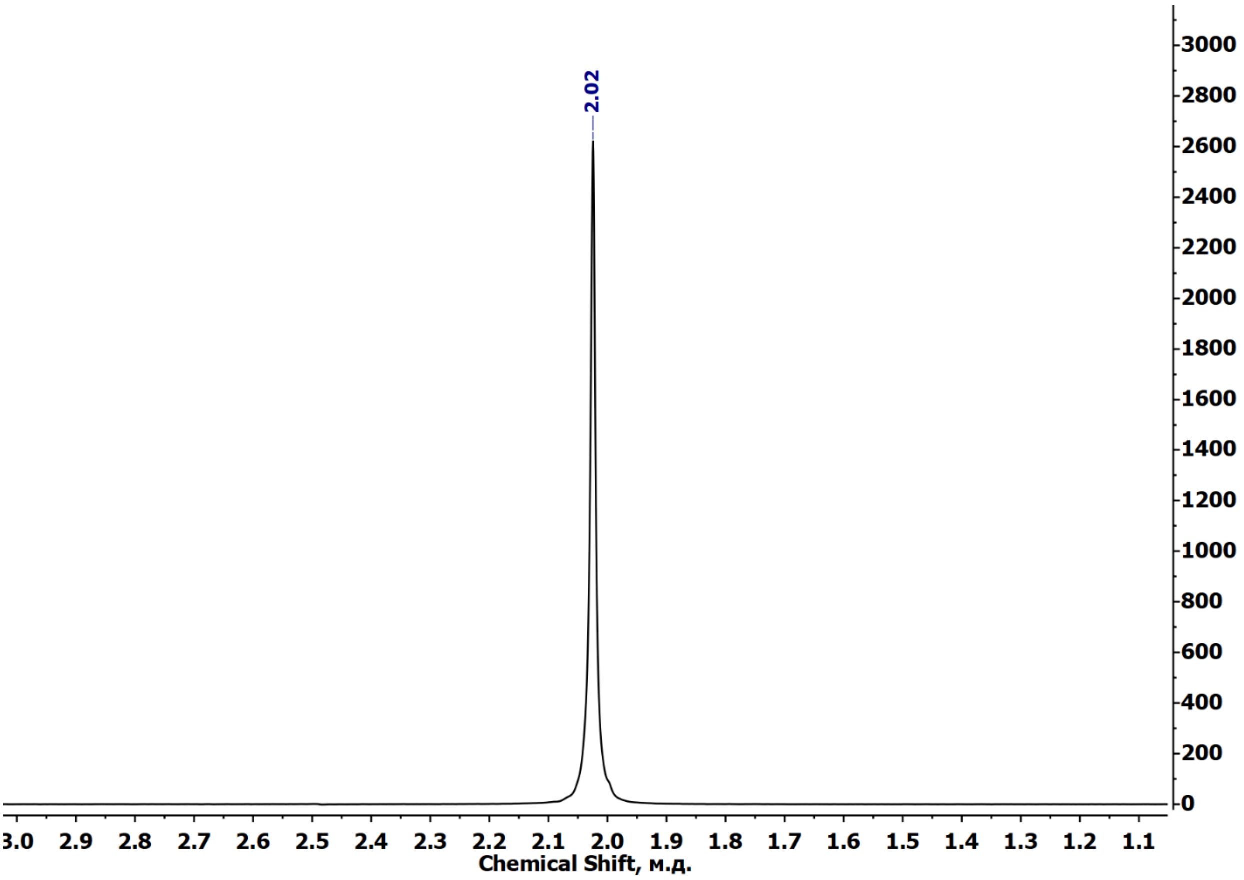

Figure S3. ^{31}P NMR INEPT spectrum of propargyl phosphate cyclohexylamine.

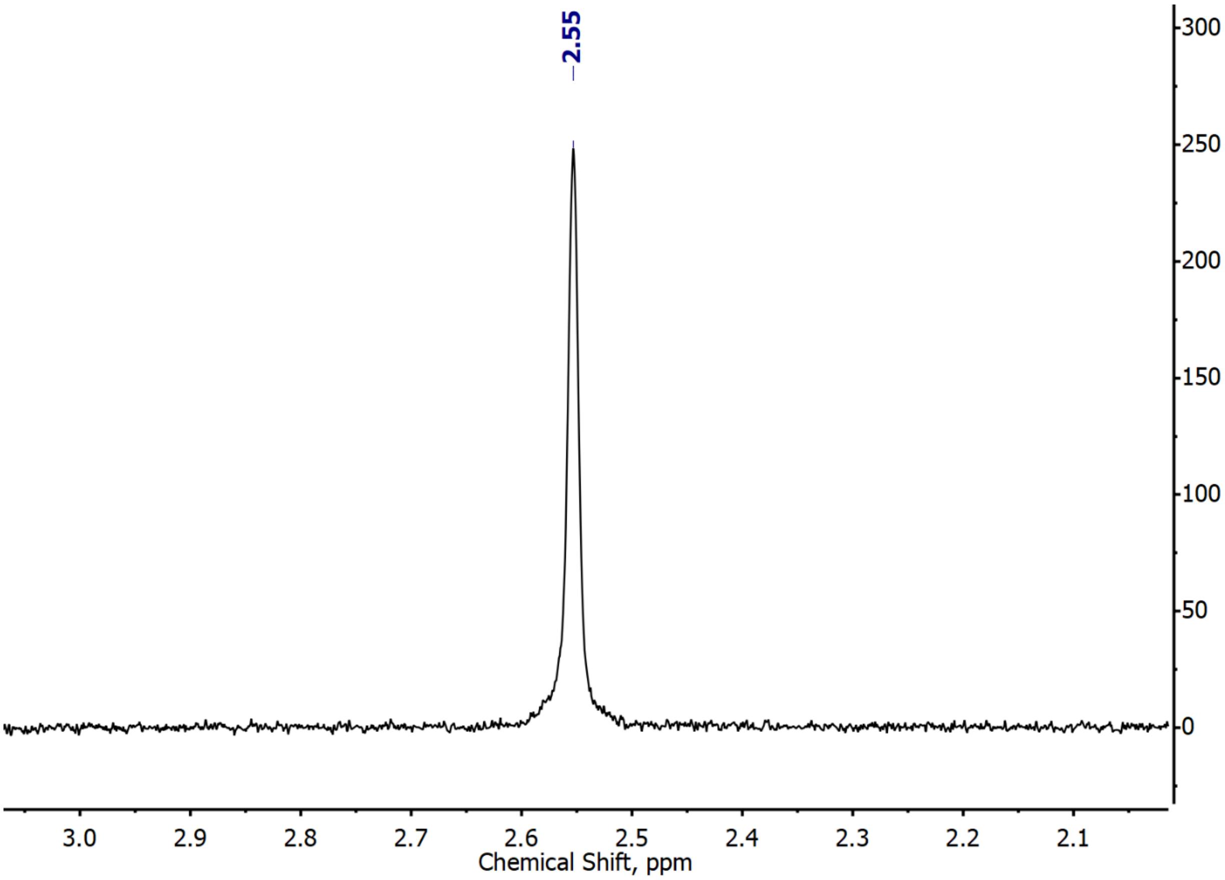

Figure S4. ^{31}P NMR spectrum of allyl phosphate cyclohexylamine.

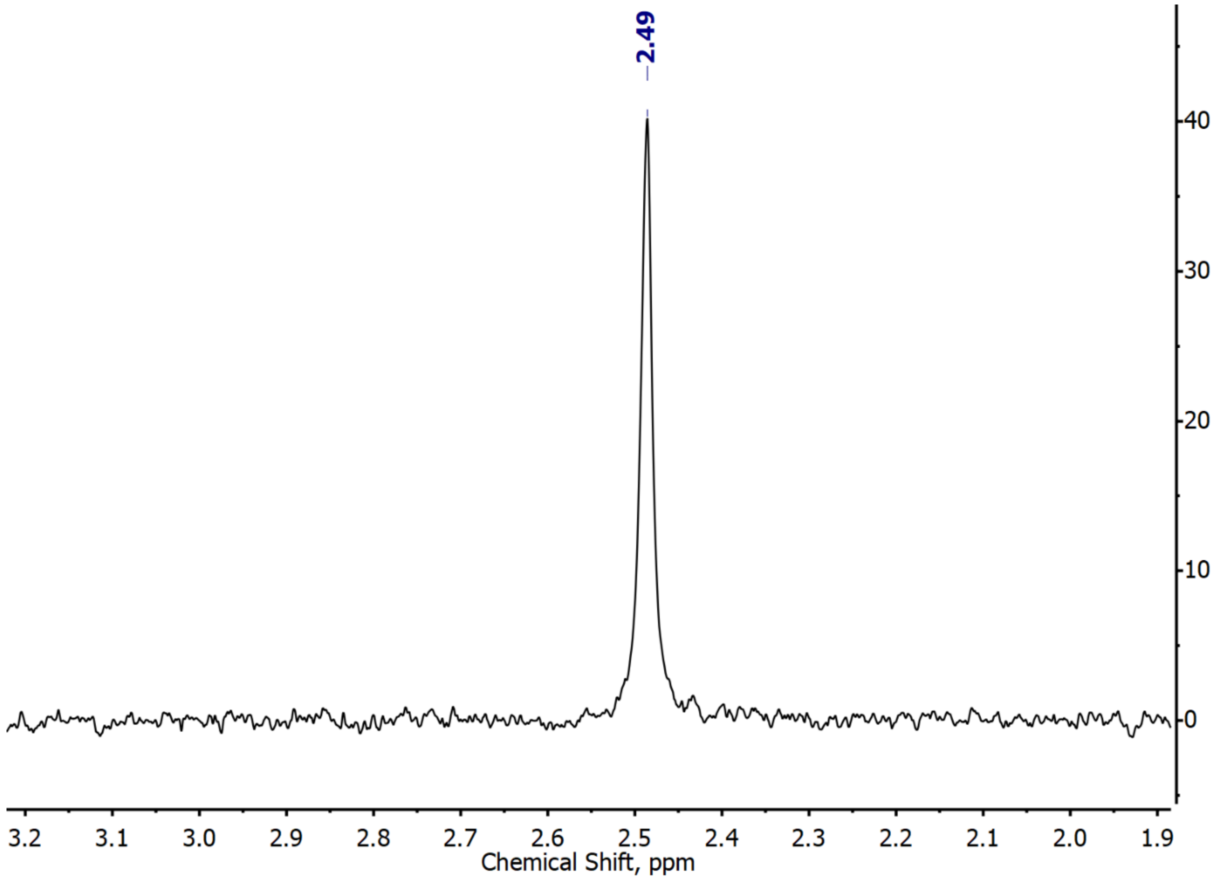

Figure S5. ^{31}P NMR spectrum of propargyl phosphate cyclohexylamine.

Supplementary Tables:

Table S1. Spin-spin scalar couplings and chemical shifts from NMR spectra of allyl phosphate and propargyl phosphate.

**Allyl phosphate**[a)]				
J coupling, Hz	CH3 (1)	CH2 (2)	CH2 (3)	P (4)
CH3 (1)		7.42	0	0
CH2 (2)			6.64	0
CH2 (3)				6.2
P (4)				
Chemical shifts, ppm	0.966	1.651	3.821	1.73

**Propargyl phosphate**[a)]						
J coupling, Hz	H1	H2	H3	H4	H5	P6
H1 (B)		1.969	17.196	1.803	1.803	0
H2 (C)			10.445	1.538	1.538	0.487
H3 (A)				5.197	5.197	0.612
H4 (D)					-15	6.000
H5 (D)						6.000
P6						
Chemical shifts, ppm	5.323	5.090	6.009	4.371	4.371	1.47

[a)]Allyl phosphate contains 7 protons and one ^{31}P nuclei while propargyl phosphate, 5 protons and one ^{31}P nuclei (Scheme S1). The appropriate NMR parameters were determined from the ^{1}H and ^{31}P NMR spectra of the products by fitting them with the program ANATOLIA [1].

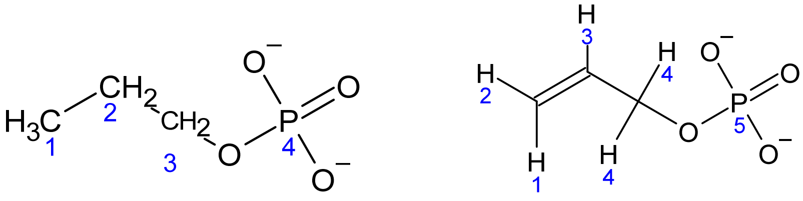

Allyl-phosphate Propargyl-phosphate

Scheme S1. Allyl-phosphate and Propargyl-phosphate.

Supplementary References:

1. Cheshkov, D.A.; Sinitsyn, D.O. Total Line Shape Analysis of High-Resolution NMR Spectra. In Annual Reports on NMR Spectroscopy; Elsevier, 2020; Vol. 100, pp. 61–96.
